# Angong Niuhuang Pill ameliorates cerebral ischemia/reperfusion injury in mice partly by restoring gut microbiota dysbiosis

**DOI:** 10.3389/fphar.2022.1001422

**Published:** 2022-09-15

**Authors:** Han Zhang, Xianrui Hui, Yule Wang, Yi Wang, Xiaoyan Lu

**Affiliations:** ^1^ Pharmaceutical Informatics Institute, College of Pharmaceutical Sciences, Zhejiang University, Hangzhou, China; ^2^ College of Pharmaceutical Science, Zhejiang Chinese Medical University, Hangzhou, China; ^3^ Hangzhou Institute of Innovative Medicine, Zhejiang University, Hangzhou, China; ^4^ Westlake Laboratory of Life Sciences and Biomedicine, Hangzhou, China

**Keywords:** Angong Niuhuang Pill, traditional Chinese medicine, cerebral ischemia/reperfusion injury, gut microbiota, metabolomics, correlation analysis

## Abstract

Angong Niuhuang Pill (ANP) is a famous traditional Chinese patent medicine and is used for treating ischemic or hemorrhagic stroke for centuries. However, the mechanism of action of ANP in stroke treatment has rarely been reported. With increasing evidence for a mechanistic link between acute ischemic stroke and gut microbiota alterations, this study aimed to determine the mechanism of action of ANP in treating acute ischemic stroke from the perspective of the gut microbiota. A mouse model of acute ischemic stroke by middle cerebral artery occlusion (MCAO) was established, and 16S ribosomal RNA (rRNA) gene sequencing and metabolomic analysis were performed on the cecal content samples collected from the sham, model, and ANP-treated MCAO mice. The results showed that ANP significantly ameliorated cerebral infarct volume, improved neurological deficits, and reduced histopathological injuries in the ipsilateral ischemic cortex, hippocampus, and striatum. The latter effects included inhibition of neuronal death, increased Nissl bodies, and decreased cell apoptosis. Moreover, ANP reversed gut microbiota dysbiosis by modulating the abundance of bacteria whose effects may mitigate MCAO damage, such as the phyla *Bacteroidetes* and *Firmicutes*, the families *Lachnospiraceae* and *Prevotellaceae*, and the genera *Alloprevotella* and *Roseburia*. Microbial metabolites related to inflammation and neuroprotection, such as prostaglandin I2 and uridine, were also regulated by ANP treatment. Uridine, guanosine, and inosine might be potential neuromodulators produced by the gut microbiota in the ANP-treated group. Spearman correlation analysis revealed that these metabolites were intimately related to certain genera, including *Alloprevotella*, *Lachnoclostridium*, *Enterorhabdus*, *Roseburia*, *Lachnospiraceae_UCG-006*, and *Colidextribacter*. Our results demonstrated that alleviating gut microbiota dysbiosis is one of the mechanisms by which ANP protects against ischemic stroke and suggest that targeting *Alloprevotella*, *Lachnoclostridium*, *Enterorhabdus*, *Roseburia*, *Lachnospiraceae_UCG-006*, and *Colidextribacter* might be a potential anti-stroke therapy.

## 1 Introduction

Stroke is a neurological deficit disease characterized by an acute focal injury to the central nervous system with a vascular cause (Campbell and Khatri, 2020). Globally, 25% of the population is affected by stroke during their lifetime (Campbell and Khatri, 2020). In China, with 2.4 million new cases and 1.1 million deaths annually (Wu et al., 2019), stroke is the leading cause of death and disability among all diseases, placing a huge burden on society. Ischemic stroke is the predominant type of stroke and accounts for 85% of all cases (Tadi and Lui, 2021). Currently, tissue plasminogen activator (tPA) is the only drug available to treat acute ischemic strokes. Nevertheless, very few patients benefit from it due to the narrow therapeutic window and the high risk of symptomatic intracranial/systemic hemorrhage (Marshall, 2015), suggesting an urgent need for new therapeutic strategies.

Growing evidence indicates that modulation of the gut microbiota is potentially a novel strategy for preventing and treating stroke (Pluta et al., 2021; Yamashiro et al., 2021). Clinical data have demonstrated that a marked dysbiosis of the gut microbiome occurs in patients with acute ischemic stroke, and the stroke dysbiosis index (SDI), which reflects the profound difference in microbial taxonomic features, can predict disease severity and early unfavorable outcome (Xia et al., 2019). This implicates the microbiota-gut-brain axis in stroke pathology. Gut microbiota can modulate brain functions through bidirectional communication along the microbiota-gut-brain axis (Martin et al., 2018). Moreover, gut microbiota-related metabolites, such as short-chain fatty acids (SCFAs) and trimethylamine N-oxide, are closely related to stroke pathogenesis and outcome (Chen et al., 2019b; Nam, 2019), suggesting that targeting gut microbiota metabolites is a novel avenue for the effective management of stroke.

Angong Niuhuang Pill (ANP), first recorded in the book of Wen Bing Tiao Bian of the Qing dynasty, is a famous traditional Chinese patent medicine for the treatment of acute cerebrovascular diseases such as ischemic and hemorrhagic stroke (Guo et al., 2014; Liu et al., 2019). This patent medicine comprises 11 types of Chinese medicinal materials, including *Bovis Calculus*, *Powdered Buffalo Horn Extract*, *Artificial Moschus*, *Margarita*, *Cinnabaris*, *Realgar*, *Coptidis Rhizoma*, *Scutellariae Radix*, *Gardeniae Fructus*, *Curcumae Radix*, and *Borneolum Syntheticum*. The main chemical constituents of ANP include flavonoids, alkaloids, and iridoid glycosides. In the clinical treatment of ischemic stroke, ANP can promote the recovery of comatose patients, improve neurological function, and reduce cerebral edema (Wu et al., 2012; Feng and Yang, 2015; Zheng et al., 2021).

Although a few studies have demonstrated that the protective effects of ANP in model rats with middle cerebral artery occlusion (MCAO) may be due to its anti-oxidative and anti-inflammatory properties (Wang et al., 2014; Tsoi et al., 2019; Zhang et al., 2021), the underlying mechanism of ANP in the treatment of stroke, especially involving gut microbiota, is seldom addressed in the literature.

Accumulating evidence suggests that traditional Chinese medicines can manage the gut microbiota and their metabolites (Feng et al., 2018; Gong et al., 2020). Moreover, several Chinese medicinal materials and components in ANP have been shown to modulate gut microbiota, such as the extracts of *Coptidis Rhizoma* and *Scutellariae Radix* (He et al., 2017; Cui et al., 2018; Xiao et al., 2020; Lyu et al., 2021; Zhao et al., 2021). It is worth exploring whether ANP exerts anti-stroke effects partly by regulating gut microbiota. Therefore, in this study, we aimed to use 16S rRNA gene sequencing coupled with metabolomics analysis to investigate the regulatory effects of ANP on the gut microbiota and their metabolites in MCAO mice.

## 2 Methods

### 2.1 Angong Niuhuang Pill preparation

ANP was provided by Huqingyutang Pharmaceutical Co., Ltd (Hangzhou, China, Batch No. 2101109). This patent medicine comprises 11 types of Chinese medicinal materials, including *Bovis Calculus* (the dry gallstones of *Bos Taurus domesticus* Gmelin), *Powdered Buffalo Horn Extract* (concentrated powder extracted from the horn of *Bubalus bubalis* Linnaens), *Artificial Moschus*, *Margarita* (*Pteria martensii* Dunker, *Hyriopsis cumingii* Lea, or *Cristaria plicata* Leach), *Cinnabaris*, *Realgar*, *Coptidis Rhizoma* (Ranunculaceae; *Coptis chinensis* Franch., *Coptis deltoidea* C. Y. Cheng et Hsiao, or *Coptis teeta* Wall.), *Scutellariae Radix* (Lamiaceae; *Scutellaria baicalensis* Georgi), *Gardeniae Fructus* (Rubiaceae; *Gardenia jasminoides* Ellis), *Curcumae Radix* (Zingiberaceae; *Curcuma wenyujin* Y. H. Chen et C. Ling, *Curcuma longa* L., *Curcuma kwangsiensis* S. G. Lee et C. F. Liang, or *Curcuma phaeocaulis* Val.), and *Borneolum Syntheticum*. The proportions of these 11 types of Chinese medicinal materials were 4:8:1:2:4:4:4:4:4:4:1. The main processing steps of ANP were as follows: *Margarita* was pulverized into very fine powders (200 mesh, >95%); *Cinnabaris* and *Realgar* were ground into very fine powders (200 mesh, >95%) and water was added; *Coptidis Rhizoma*, *Scutellariae Radix*, *Gardeniae Fructus*, and *Curcumae Radix* were pulverized into fine powders (100 mesh, >95%); *Bovis Calculus*, *Powdered Buffalo Horn Extract*, *Artificial Moschus*, and *Borneolum Syntheticum* were ground with the above powders, then sieved and well mixed; The mixture was then mixed with refined honey and prepared as 600 large honeyed pills. In this study, the ANP was dissolved in ultrapure water before intragastric administration. High-performance liquid chromatography analysis of ANP was performed and the main constituents in ANP were geniposide, baicalin, berberine, wogonoside, baicalein, wogonin, and oroxylin A (Supplementary Figure S1). The detailed description of ANP sample preparation and high-performance liquid chromatography parameters is provided in the Supplementary Materials.

### 2.2 Animals

Male C57BL/6 mice (6–9 weeks old, 18–24 g) were obtained from Beijing Vital River Lab Animal Technology Co. Ltd. (Beijing, China) and acclimatized at Zhejiang Chinese Medical University Laboratory Animal Research Center. All mice had free access to food and water and were housed under constant temperature (24 ± 1°C) and humidity (55% ± 10%) with light and dark conditions alternating every 12 h. All procedures were approved by the Institutional Animal Care and Use Committee of Zhejiang Chinese Medical University.

### 2.3 Groups and drug administration

Because the common human daily dose of ANP is 3 g/60 kg/day, the equivalent dose for mice was 450 mg/kg/day according to the Meeh–Rubner equation. The mice were acclimatized for at least 7 days and then randomly divided into three groups: sham, model (MCAO), and ANP (MCAO + ANP). The mice in the ANP group were intragastrically administered ANP at a dosage of 450 mg/kg/day for seven consecutive days, while those in the sham and model groups were administered the same quantity per day of ultrapure water (20 ml/kg). In this study, n was set to 32 in each group, of which 24 mice were used for gut microbiota sequencing and metabolomic analyses, five mice were used for 2,3,5-triphenyltetrazolium chloride (TTC) staining, and the remaining three mice were used for histopathological test.

### 2.4 Middle cerebral artery occlusion procedure

The MCAO procedure was performed 1 h after the last administration based on a previously established protocol (Xu et al., 2021). Briefly, the mice were fasted for 12 h before MCAO and then anesthetized with sodium pentobarbital (1%, 8 ml/kg) by intraperitoneal injection. Subsequently, the left common carotid artery (CCA), external carotid artery (ECA), and internal carotid artery (ICA) were carefully exposed, and an L1800 silicone filament (Guangzhou Jialing Biotechnology Co., Ltd., Guangzhou, China) was inserted into the ICA and lodged in the origin of the middle cerebral artery (MCA) to induce MCAO. After 90 min, the filament was withdrawn to enable MCA recanalization. Mice in the sham group underwent the same procedure, except for MCA occlusion and recanalization. The mice were excluded from analysis if any of the following occurred: underweight before the procedure (<20 g), vascular anomalies found, or death occurred during or after the MCAO procedure. The neurological deficit score was assessed 24 h after reperfusion based on the Zea–Longa scoring criteria (Longa et al., 1989). Following this, the brains and cecal contents were obtained from anesthetized mice for subsequent analyses.

### 2.5 Measurement of cerebral infarct volume

Brains were cut into 5 thick slices and stained with 0.2% TTC (Sigma, St. Louis, MO, United States). After immersion in 4% paraformaldehyde, the slices were photographed, and the cerebral infarct volume was measured using ImageJ (version 1.53).

### 2.6 Hematoxylin-eosin, Nissl, and terminal deoxynucleotidyl transferase dUTP nick end labeling staining

After immersion in 4% paraformaldehyde, the brains were dehydrated and embedded in paraffin for coronal sectioning. The coronal sections were used for Hematoxylin-eosin (HE) staining, Nissl staining, and terminal deoxynucleotidyl transferase dUTP nick end labeling (TUNEL) staining. The sections were dewaxed, hydrated, and stained with HE. For Nissl staining, sections were dewaxed, hydrated, and stained with 1% toluidine blue; for TUNEL staining, sections were analyzed using a TUNEL assay kit (Roche, Basel, Switzerland). Finally, each section was observed using an Olympus VS200 system (Olympus, Tokyo, Japan). The percentage of TUNEL positive cells was quantified using ImageJ (version 1.53). The total and TUNEL positive cell numbers were counted in three different regions of each section.

### 2.7 Gut microbiota sequencing and data analysis

To reduce the effect of individual differences, equal amounts of cecal contents from the same group of four mice were pooled and used as a representative sample for that group. Six mixed samples from 24 mice from each group were used for gut microbiota sequencing. Bacterial DNA was isolated from cecal contents using a MagPure Soil DNA LQ Kit (Guangzhou Magen Biotechnology Co., Ltd., Guangzhou, China). Then, the V3-V4 variable regions of the bacterial 16S rRNA were amplified with universal primers after DNA quantification. The PCR products were purified and quantified before sequencing. After library construction, metagenomic sequencing was conducted using a NovaSeq 6000 platform (Illumina, San Diego, CA, United States). Raw sequence data were uploaded to the Sequence Read Archive (SRA) database of the National Center for Biotechnology Information (NCBI) and linked to bioproject PRJNA855110.

Trimmomatic (version 0.35) was used to delete low-quality sequences (Bolger et al., 2014), and FLASH (version 1.2.11) was applied to construct paired-end reads (Reyon et al., 2012). Clean reads were then obtained by de-noising with QIIME (version 1.8.0) (Caporaso et al., 2010), and effective reads were acquired using UCHIME (version 2.4.2) (Edgar et al., 2011). These were used to generate operational taxonomic units with Vsearch (version 2.4.2) (Rognes et al., 2016). All representative reads were annotated using the RDP classifier in the SILVA database (version 138) (Wang et al., 2007; Quast et al., 2013).

The Simpson index was used to compare alpha diversity. Principal coordinates analysis (PCoA) was performed using the Bray-Curtis dissimilarity metric to compare beta diversity. The SDI was calculated as previously described with minor modifications (Xia et al., 2019). First, a Wilcoxon rank-sum test was performed to detect significant differences between the sham and model groups at the genus level. A *p* value <0.05 was set as the threshold to select differential genera. Finally, Eq. 1 was used to calculate the SDI.

$$SDI=\left\{\frac{\sum_{i=7}relative\;abundance\;\left(Model\;enriched\right)i}{7}-\frac{\sum_{j=8}relative\;abundance\;\left(sham\;enriched\right)j}{8}\right\}\times 100$$ (1)

The linear discriminant analysis (LDA) effect size (LEfSe) method was used to determine gut microbial markers among the groups. T-test analysis was used for statistical significance comparison between the two groups at the phylum, family, and genus levels. The Wilcoxon rank-sum test was used to calculate the statistical significance of differences in the Simpson index and the SDI between the two groups.

### 2.8 Metabolomics analysis

Similarly, equal amounts of cecal contents from the same group of four mice were pooled and six mixed samples from 24 mice from each group were analyzed by liquid or gas chromatography–mass spectroscopy (LC/GC-MS).

#### 2.8.1 Liquid chromatography–mass spectroscopy

Sample preparation for LC-MS analysis was conducted as follows: Briefly, the metabolites in the cecal content sample (60 mg) were extracted with methanol/water (4/1, v/v). L-2-chlorophenylalanine was added as an internal standard during sample processing. The metabolites were filtered through a 0.22 μm microfilter and used for LC-MS analysis. Quality control (QC) sample was prepared by mixing equal aliquots of all samples.

LC-MS analysis was performed on an ACQUITY UPLC HSS T3 column (100 × 2.1 mm, 1.8 μm) (Waters Corporation, Milford, MA, United States) using an ACQUITY UPLC I-Class system (Waters Corporation) combined with a QE plus mass spectrometer (Thermo Scientific, Waltham, MA, United States). The detailed parameters for the liquid chromatography and mass spectrometry procedures are provided in the Supplementary Materials. The QC sample was injected three times during the entire course of the analysis.

The raw LC-MS data were preprocessed using Progenesis QI (version 2.3). The extracted data were further processed by removing the peaks detected in less than half of the group samples or those with a relative standard deviation (RSD) > 30% in the QC samples. The missing values were increased by half of the minimum values. For compound identification, we used the Human Metabolome Database (HMDB) (Wishart et al., 2007), the LIPID MAPS database (version 2.3) (Fahy et al., 2007), and the Metlin database (Smith et al., 2005). The detected compounds were verified manually. The LC-MS data for the positive and negative ion modes were combined for subsequent analysis.

Principal component analysis (PCA) was used for quality evaluation of the LC-MS data. Orthogonal partial least-squares-discriminant analysis (OPLS-DA) and *t*-test analysis were utilized to identify the metabolites differentially expressed between groups, and the variable important in projection (VIP) > 1 and *p* value <0.05 were set as the threshold.

#### 2.8.2 Gas chromatography–mass spectroscopy

Sample preparation for GC-MS analysis was conducted as follows: Briefly, the metabolites in the cecal content sample (60 mg) were extracted with methanol, and chloroform/water (1/2, v/v). The metabolites were then oximated and derivatized. L-2-chlorophenylalanine and ten fatty acid methyl esters (C8/C9/C10/C12/C14/C16/C18/C20/C22/C24) were added as internal standards during sample processing. After storage at room temperature for 30 min, the metabolites were analyzed by GC-MS. The QC sample was prepared by mixing equal aliquots of all samples.

GC-MS analysis was executed on a DB-5MS fused-silica capillary column (30 m × 0.25 mm × 0.25 μm) (Agilent, Santa Clara, CA, United States) using an Agilent 7890B gas chromatography system combined with a 5977B MSD system (Agilent). The detailed parameters for the gas chromatography and mass spectrometry procedures are provided in the Supplementary Materials. The QC sample was injected five times during the entire course of the analysis.

The raw GC-MS data were preprocessed using MS-DIAL (Tsugawa et al., 2015). A self-built database (Luming Biotech CO., LTD., Shanghai, China) was used for compound identification. The detected compounds were verified manually. The missing values were increased by half of the minimum values. For each sample, all peak signal intensities were segmented and normalized to the internal standards, with RSD < 30% after screening.

Principal component analysis (PCA) was used for quality evaluation of the GC-MS data. OPLS-DA and *t*-test analyses were used to identify the metabolites differentially expressed between groups with VIP >1 and *p* value <0.05.

### 2.9 Statistical analysis

All data are reported as mean ± standard error of the mean. Statistical differences were examined using the *t*-test, the Wilcoxon rank-sum test, or one-way ANOVA, and correlations were evaluated using Spearman’s test. The data were analyzed using GraphPad Prism (version 8.0.1), SPSS (version 25), or OECloud tools (https://cloud.oebiotech.cn.). Plotting was performed using GraphPad Prism (version 8.0.1), R software (version 3.5.1), or OECloud tools.

## 3 Results

### 3.1 Angong Niuhuang Pill ameliorated cerebral infarct volume and the neurological deficits

**FIGURE 1:**
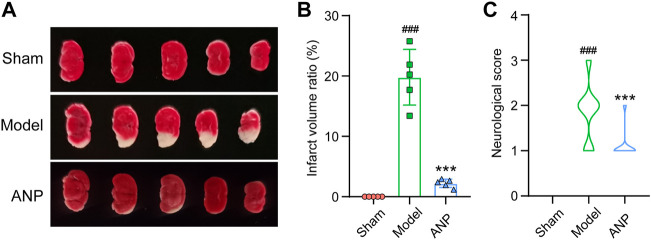
ANP reduced the infarct volume ratio and the neurological deficit score. **(A)** The representative images of TTC-staining. **(B)** The infarct volume ratio (*n* = 5). Statistical differences were examined by one-way ANOVA. **(C)** The neurological score (*n* = 32). Statistical differences were examined by one-way ANOVA. ^###^ *p* < 0.001 compared with the sham group; ^***^ *p* < 0.001 compared with the model group.

To verify the effects of ANP on acute ischemic stroke, infarct size and neurological deficit score were assessed 24 h after cerebral ischemia/reperfusion in mice. Representative images of TTC-staining are displayed in Figure 1A. Quantitative data on the infarct volume ratio are shown in Figure 1B. The brain tissues of the sham group showed a red color with no infarction, while those of the model group exhibited a pale color in the infarcted hemisphere, with an average infarct volume ratio of 20%. ANP treatment significantly ameliorated the focal infarction volume, with the ratio decreasing to 2%. Similar trends were observed in the neurological deficit scores, where ANP administration markedly mitigated the neurological deficits induced by the MCAO model (Figure 1C). These results suggest that treatment with ANP alleviates cerebral ischemia/reperfusion injury.

### 3.2 Angong Niuhuang Pill reduced the histopathological injuries

**FIGURE 2:**
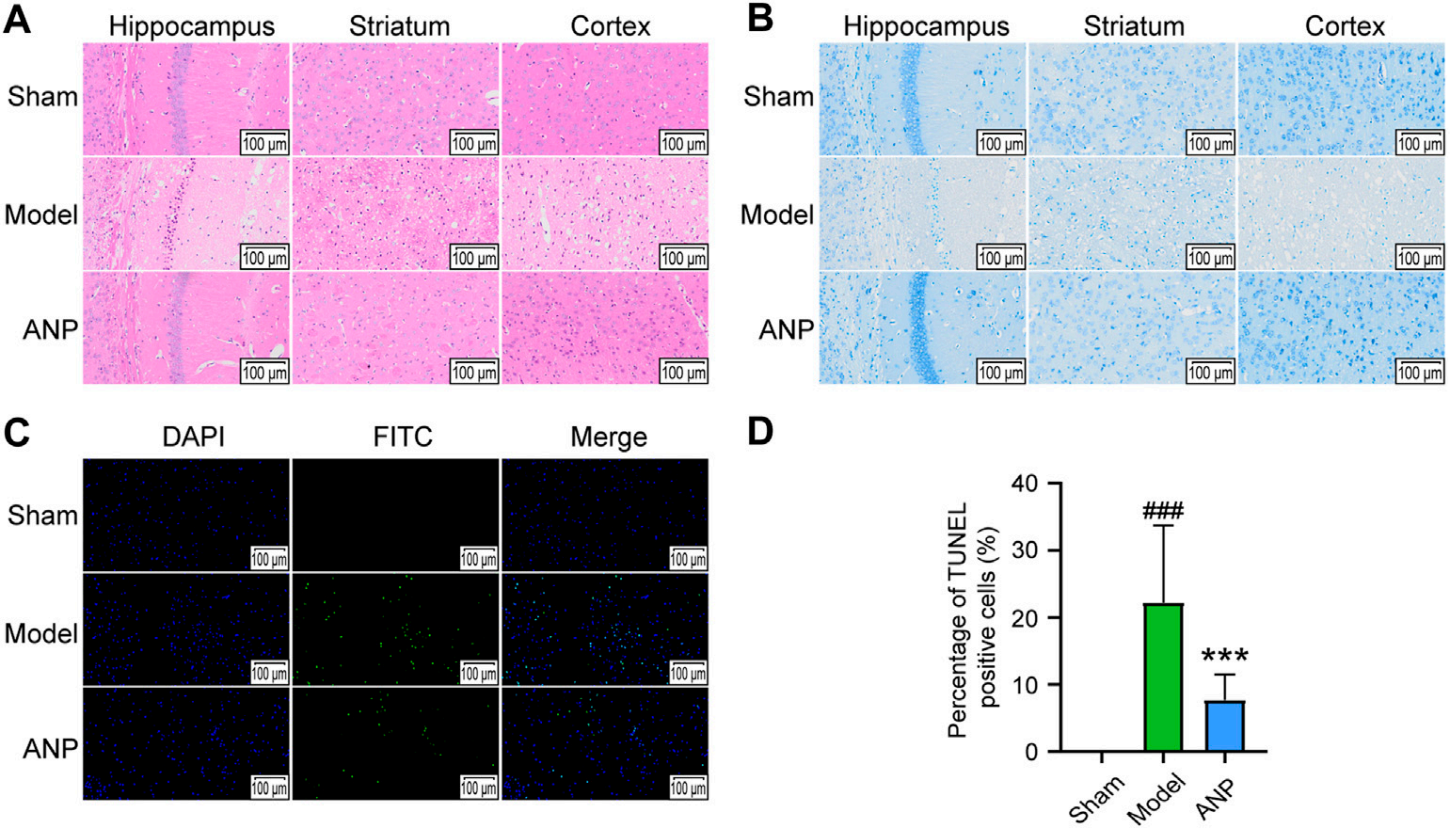
ANP alleviated the pathological injury. **(A)** The representative images of HE staining (*n* = 3). **(B)** The representative images of Nissl staining (*n* = 3). **(C)** The representative images of TUNEL staining (*n* = 3). **(D)** The percentage of TUNEL positive cells. Magnification of 300 ×. Statistical differences were examined by one-way ANOVA. ^###^ *p* < 0.001 compared with the sham group; ^***^ *p* < 0.001 compared with the model group.

As shown in Figure 2A, HE staining revealed that the sham group had normal neurons in the cortex, hippocampus, and striatum. However, the mice in the model group exhibited numerous dead neurons showing nuclear shrinkage and vacuoles in the ipsilateral ischemic cortex, hippocampus, and striatum. Compared with the model group, MCAO mice that received ANP had fewer dead neurons in these regions. Similar trends were detected by Nissl staining (Figure 2B). Specifically, the sham mice displayed visible cytoplasmic Nissl bodies in the ipsilateral hemisphere, while the model group had fewer Nissl bodies in the cytoplasm; ANP treatment increased the Nissl bodies in the ipsilateral ischemic cortex, hippocampus, and striatum. Furthermore, TUNEL staining showed a consistent trend as the number of apoptotic cells were increased in the mice subjected to MCAO, and this phenomenon was inhibited by ANP administration (Figure 2C). Compared with the model group, the percentage of TUNEL positive cells of the ANP group decreased from 22% to 8% (Figure 2D). All these data indicated that treatment with ANP improved the pathological injuries caused by cerebral ischemia and reperfusion.

### 3.3 Angong Niuhuang Pill reversed middle cerebral artery occlusion-induced gut microbiota dysbiosis

To test our hypothesis that ANP exerts anti-stroke effects partly by regulating the composition of the gut microbiota, we performed metagenomics sequencing of the cecal contents from sham, model, and ANP-treated MCAO mice. The cecal content samples produced an average of 74,150 clean reads and 65,553 effective reads per sample. We identified an average of 3,236 operational taxonomic units per sample, implying that the sequencing depth was sufficient.

**FIGURE 3:**
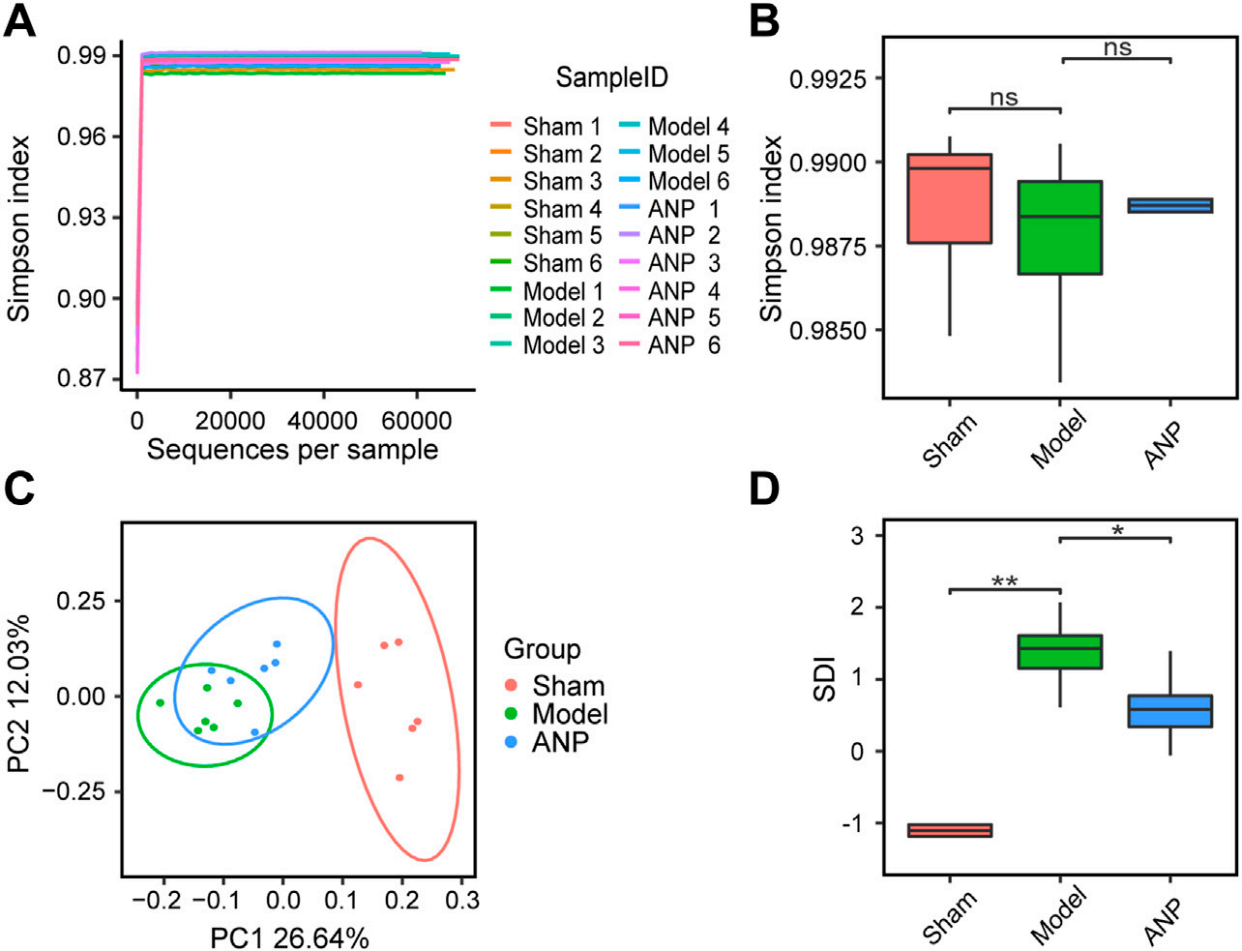
ANP reversed MCAO-induced gut microbiota dysbiosis. **(A)** Rarefaction curve analysis based on the Simpson index. **(B)** Comparison of alpha diversity based on the Simpson index. Statistical differences were examined by Wilcoxon rank-sum test. **(C)** The PCoA score plot using Bray-Curtis dissimilarity metric. **(D)** Comparison of SDI. Statistical differences were examined by Wilcoxon rank-sum test. ^*^ *p* < 0.05, ^**^ *p* <0.01, ns, no statistical difference.

Rarefaction curve analysis based on the Simpson index showed that the sequencing depth covered most of the diversity (Figure 3A). However, we observed no statistical differences in the Simpson index between the sham, model, and ANP-treated groups (Figure 3B). To compare the overall microbiota structure, a PCoA was performed based on the Bray-Curtis dissimilarity. The results showed that the samples from the model group reached a maximum shift relative to the sham group and that the samples from the ANP group were located between those of the sham and model groups, indicating that ANP reversed MCAO-induced gut microbiota dysbiosis (Figure 3C). Furthermore, we adopted the SDI to compare the differences in microbial taxonomic features among the three groups. Clinical studies show that the SDI is an indicator of stroke severity and outcome (Xia et al., 2019). Consistent with clinical data, the SDI of the model group was significantly greater than that of the sham group. ANP treatment significantly decreased the SDI compared with the model group (Figure 3D), suggesting that ANP can improve MCAO-induced gut microbiota dysbiosis.

### 3.4 Angong Niuhuang Pill regulated gut microbiota at the phylum, family, and genus levels

At the phylum level, *Bacteroidota* and *Firmicutes* were the two most dominant phyla in the three groups (Supplementary Figure S2A). Further analysis demonstrated that compared to the sham group, MCAO modeling significantly increased the relative abundance of *Bacteroidota* but reduced the abundance of *Firmicutes* (Supplementary Figure S2B). ANP significantly reversed these changes, indicating that the treatment improved gut microbiota dysbiosis at the phylum level (Supplementary Figure S2B).

The main families identified were *Muribaculaceae*, *Lachnospiraceae*, and *Prevotellaceae* (Supplementary Figure S3A). MCAO modeling significantly increased the relative abundance of *Prevotellaceae* while reducing the abundance of *Lachnospiraceae* compared to the sham group (Supplementary Figure S3B). ANP treatment significantly reversed these changes (Supplementary Figure S3B).

**FIGURE 4:**
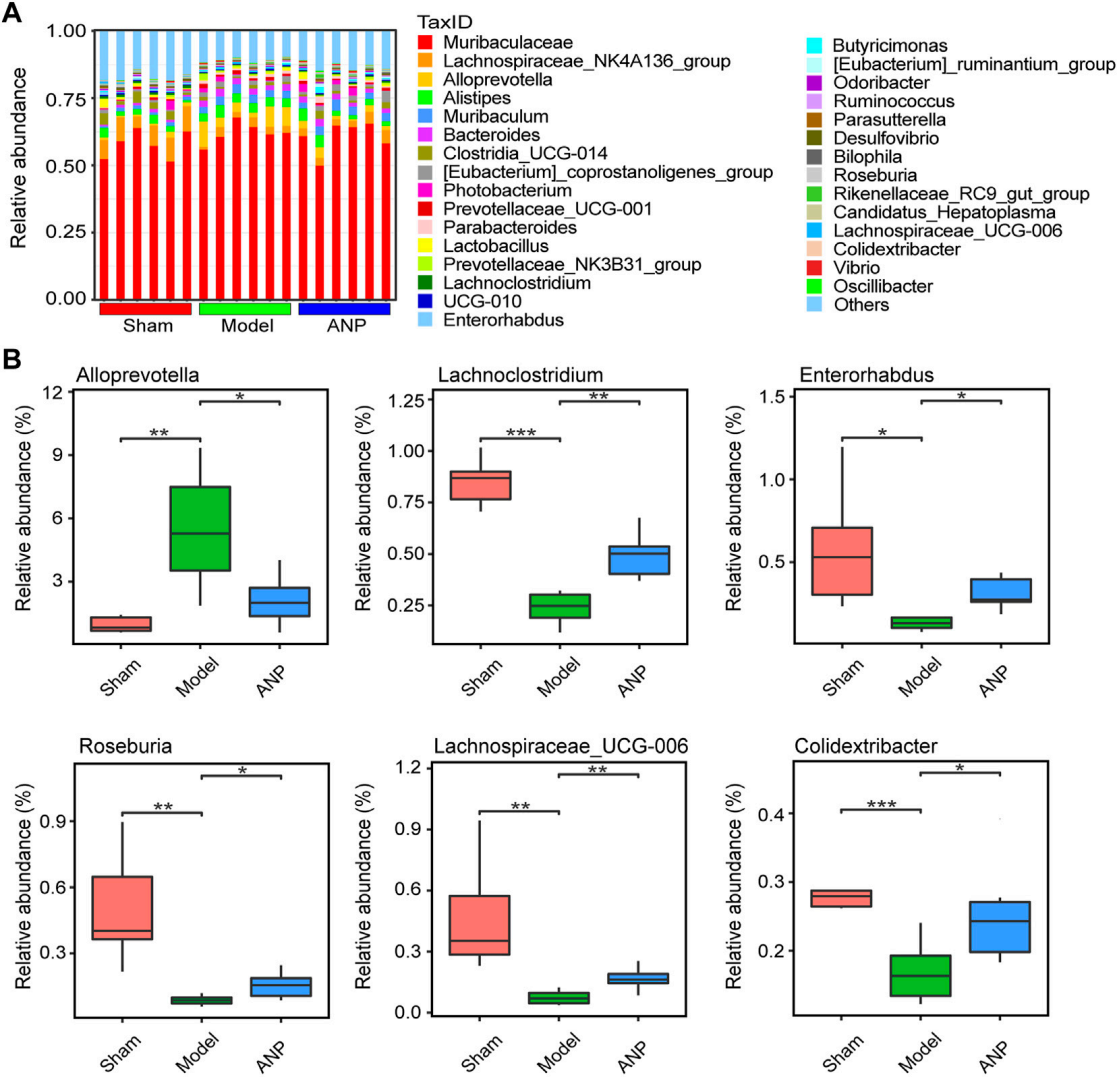
ANP regulated the gut microbiota at the genus level. **(A)** The gut microbiota composition at the genus level. **(B)** The statistical analysis at the genus level. Statistical differences were examined by *t*-test. ^*^ *p* < 0.05, ^**^ *p* < 0.01, ^***^ *p* < 0.001.

A histogram of the top-ranked genera is shown in Figure 4A. At the genus level, MCAO modeling significantly affected the relative abundance of certain genera, some of which could be renormalized by ANP. We observed a significant increase in the abundance of *Alloprevotella* and a significant decrease in the abundance of *Lachnoclostridium*, *Enterorhabdus*, *Roseburia*, *Lachnospiraceae_UCG-006*, and *Colidextribacter* in the model compared to the sham mice (Figure 4B). Notably, compared with the model group, ANP decreased the relative abundance of *Alloprevotella* and markedly increased the abundance of *Lachnoclostridium*, *Enterorhabdus*, *Roseburia*, *Lachnospiraceae_UCG-006*, and *Colidextribacter* (Figure 4B).

**FIGURE 5:**
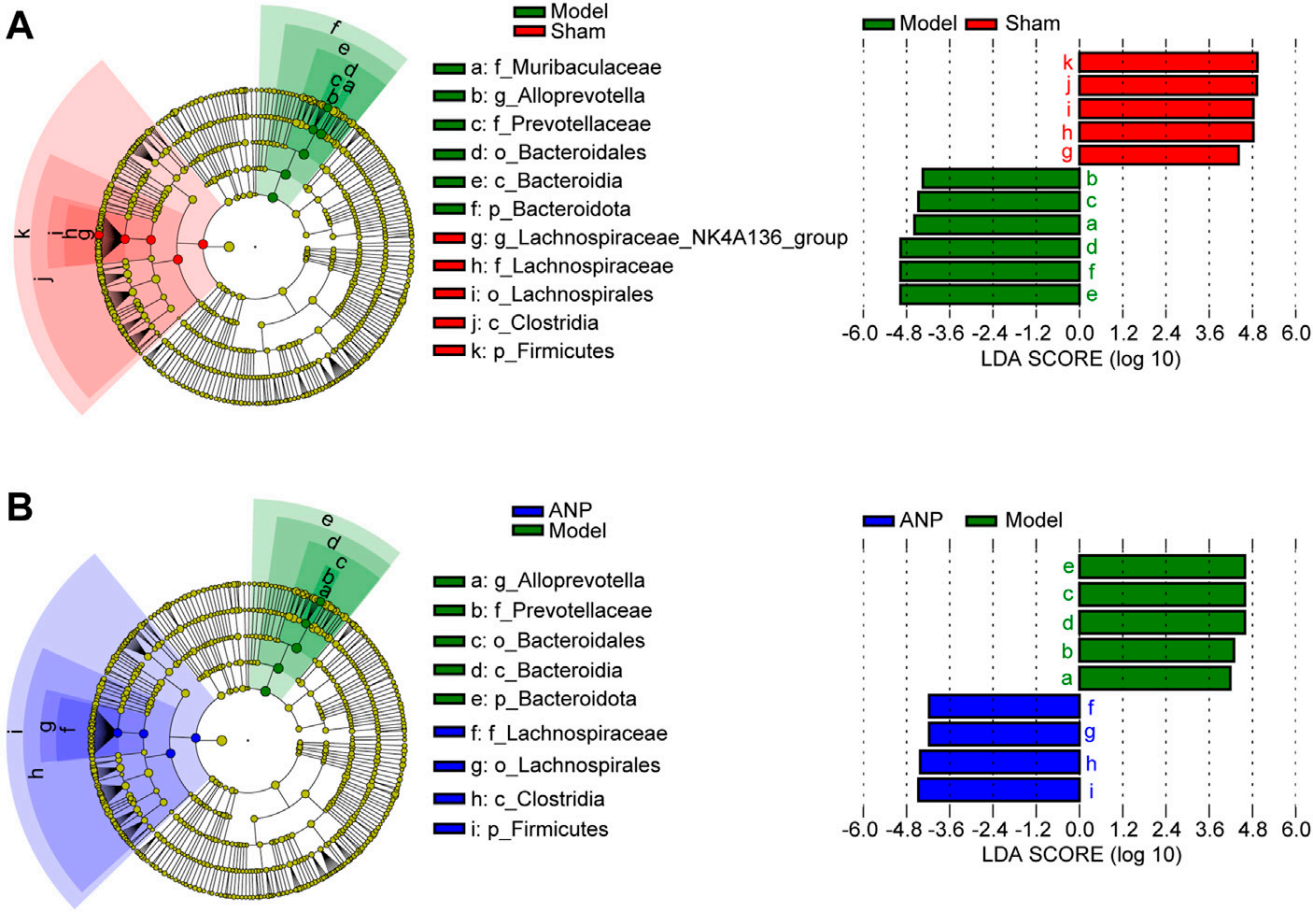
LEfSe analysis for the gut microbiota alterations among the groups. **(A)** LEfSe analysis between the model group and the sham group. The inner to outer rings represent phylum, class, order, family, and genus. **(B)** LEfSe analysis between the ANP group and the model group.

Additionally, we employed LEfSe analysis to determine the specific bacteria that differed among the sham, model, and ANP-treated groups. Figure 5 shows these results on a cladogram. The inner to outer rings represent phylum, class, order, family, and genus. An LDA score greater than 4.0 was used to identify significantly discriminative features. *Lachnospiraceae*, *Lachnospirales*, *Clostridia*, and *Firmicutes* were the dominant bacteria in both the sham and ANP groups, whereas *Alloprevotella*, *Prevotellaceae*, *Bacteroidales*, *Bacteroidia*, and *Bacteroidota* were the dominant bacteria in the model group compared to the sham and ANP groups (Figure 5). Therefore, three dominant bacteria in the model group, *Alloprevotella*, *Prevotellaceae,* and *Bacteroidota,* might be the target bacteria responsible for the therapeutic effects of ANP treatment (Supplementary Figures S2B, S3B; Figure 4B).

### 3.5 Angong Niuhuang Pill modulated the fecal metabolomic profile

**FIGURE 6:**
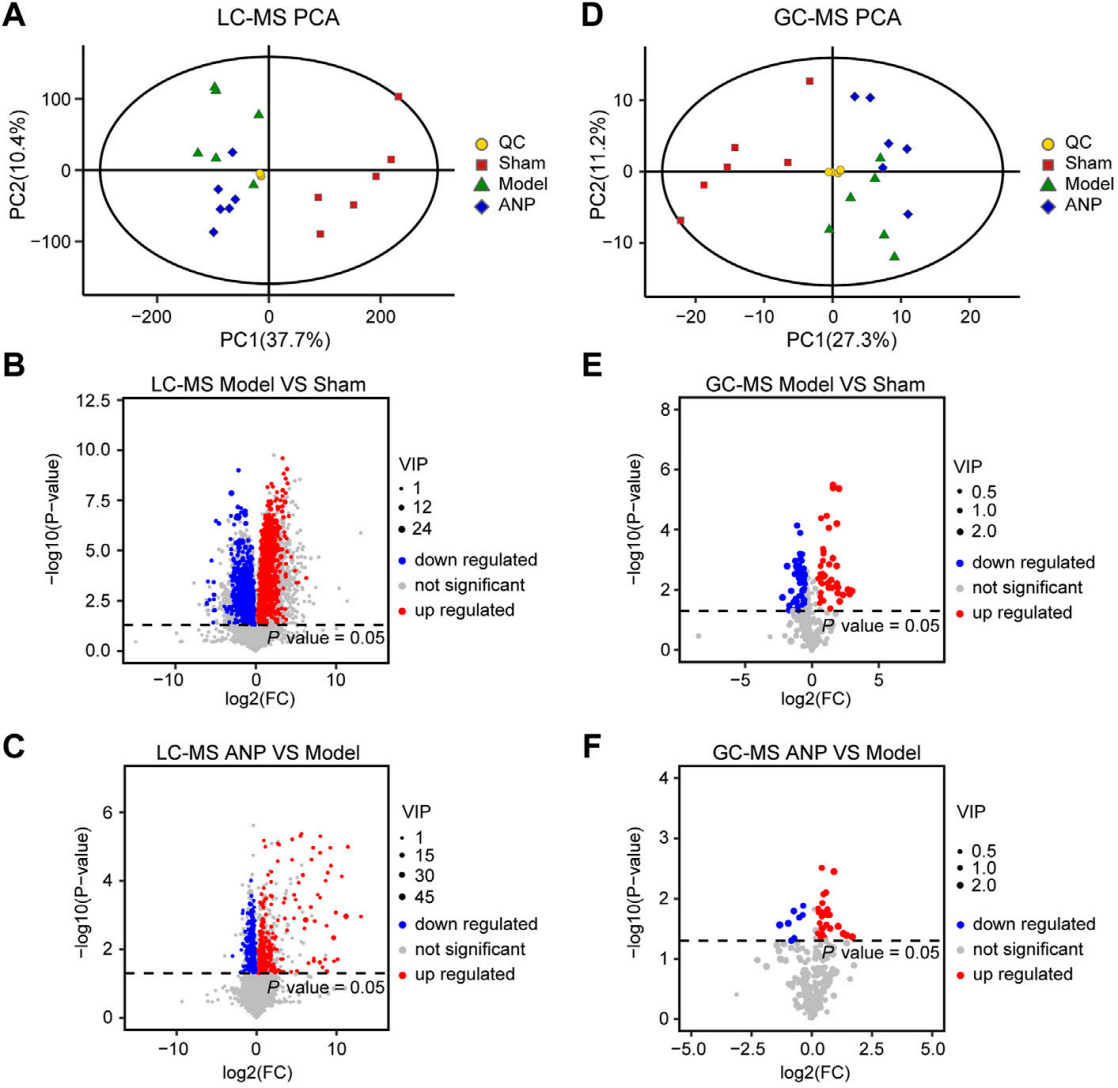
The multivariate data analysis from LC-MS and GC-MS data. **(A)** The PCA score plot of the LC-MS data. **(B,C)** The volcano plot for the differentially expressed metabolites between the model and the sham groups **(B)** as well as between the ANP and the model groups **(C)** based on the LC-MS data. **(D)** The PCA score plot of the GC-MS data. **(E,F)** The volcano plot for differentially expressed metabolites between the model and the sham groups **(E)** as well as between the ANP and the model groups **(F)** based on the GC-MS data.

LC-MS- and GC-MS-based metabolomic analyses were carried out to characterize metabolic alterations in the gut microbiota responsible for ANP treatment. The PCA score plots of the LC-MS and GC-MS data are shown in Figures 6A,D, respectively. The QC samples overlapped, indicating that the instrument precision and sample stability were adequate. The model group was separated from the sham group, suggesting metabolite changes in the gut microbiota after MCAO. While the ANP and model groups were close, distinct clusters appeared, and the ANP group exhibited a trend toward the sham group. These results suggest that ANP tended to renormalize fecal metabolome disturbed by MCAO.

**FIGURE 7:**
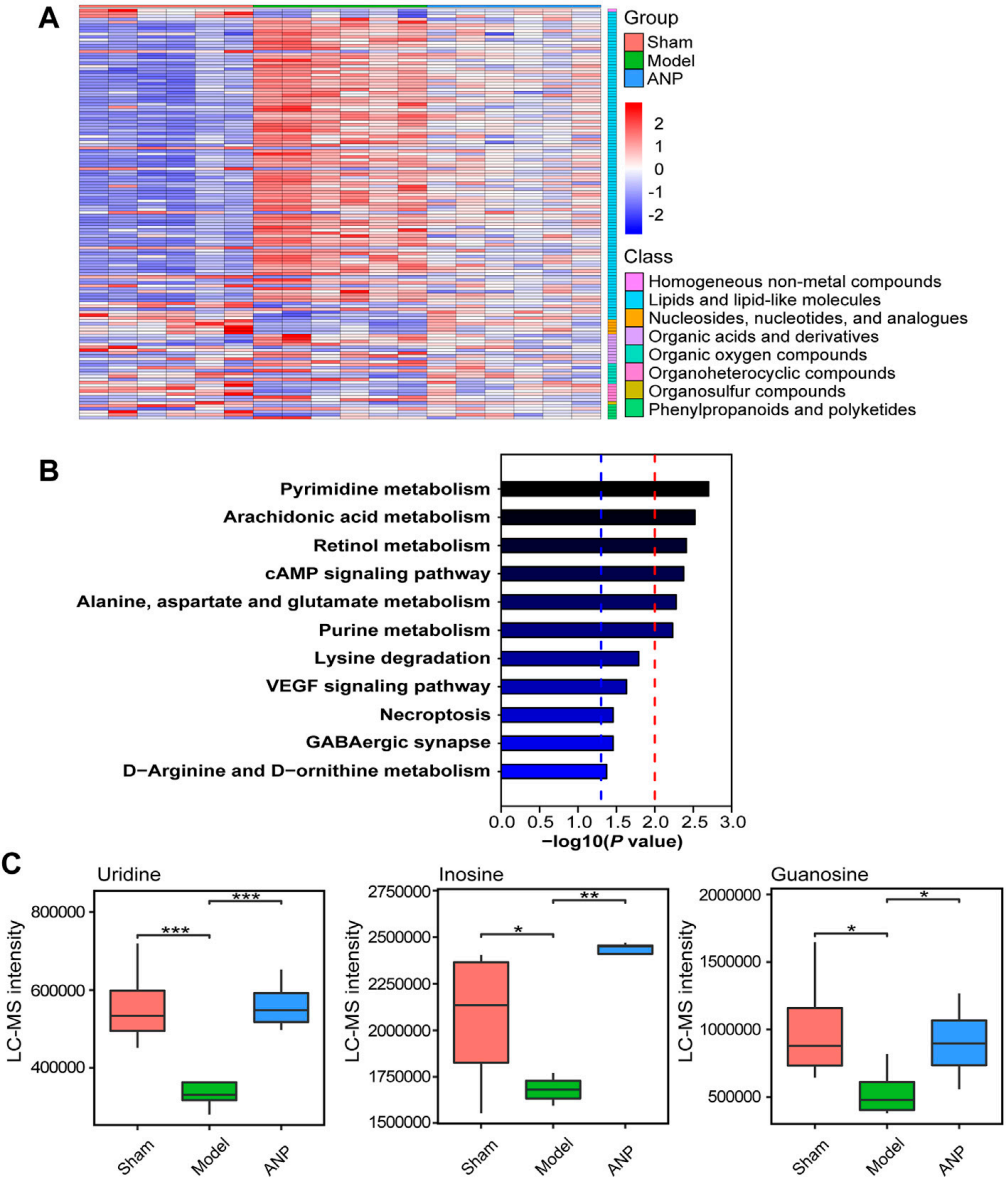
The KEGG metabolic pathway analysis of the metabolites reversed by ANP. **(A)** The heatmap of the metabolites reversed by ANP (values were log10-transformed). **(B)** The KEGG metabolic pathways. The blue dotted line represents -log10 (*p* value) = 1.3; the red dotted line represents -log10 (*p* value) = 2.0. **(C)** The statistical analysis of the key differentially expressed metabolites. Statistical differences were examined by *t*-test. ^*^ *p* < 0.05, ^**^ *p* < 0.01, ^***^ *p* < 0.001.

Then, OPLS-DA and *t*-test analyses were performed to identify differential metabolites. Plots of the OPLS-DA scores of the LC-MS and GC-MS data are shown in Fig. S4. The score plots presented a distinct clustering of metabolites in the cecal content samples between the model and sham groups, as well as between the ANP and model groups, suggesting that both MCAO and ANP influenced the metabolic profiles. Additionally, volcano plots clearly showed obvious metabolic differences between the model and sham groups and between the ANP and model groups (Figures 6B,C,E,F). Finally, 140 differentially expressed metabolites were identified using LC-MS and GC-MS analyses. The levels of 140 metabolites were deregulated by MCAO and could be reversed by ANP (Figure 7A). Detailed information on these metabolites is listed in Supplementary Tables S1, S2, including name, class, retention time, ion mode, *m/z*, and the alterations among the sham, model, and ANP-treated groups.

The Kyoto Encyclopedia of Genes and Genomes (KEGG) database was used to link the significantly differentially expressed metabolites to metabolic pathways. A total of 11 metabolic pathways (*p* < 0.05) were identified (Figure 7B), and six metabolic pathways were significantly enriched (−log_10_ *p* value > 2), including pyrimidine metabolism; arachidonic acid metabolism; retinol metabolism; cAMP signaling pathway; alanine, aspartate, and glutamate metabolism; and purine metabolism. In total, 15 metabolites were associated with these 11 metabolic pathways and were considered the key metabolites regulated by ANP (Table 1). Moreover, the variation of the mass spectrum intensities of these 15 metabolites in the three groups suggested that ANP treatment could reverse the disorder of gut microbiota metabolism induced by MCAO (Figure 7C; Supplementary Figure S5).

**TABLE 1:** The key metabolites related to the pathways.

Pathway name	Related metabolites
Pyrimidine metabolism	Cytosine, uridine, ureidosuccinic acid
Arachidonic acid metabolism	6-Keto-PGF1alpha, 20-hydroxy-leukotriene B4, prostaglandin I2
Retinol metabolism	4-Oxo-retinoic acid, 4-hydroxyretinoic acid
cAMP signaling pathway	Succinic acid, prostaglandin I2
Alanine, aspartate and glutamate metabolism	Succinic acid, ureidosuccinic acid
Purine metabolism	Guanosine, adenine, inosine
Lysine degradation	Succinic acid, L-2-hydroxyglutaric acid
VEGF signaling pathway	Prostaglandin I2
Necroptosis	SM(d18:0/20:2(11Z, 14Z))
GABAergic synapse	Succinic acid
D−Arginine and D−ornithine metabolism	Ornithine

### 3.6 Angong Niuhuang Pill regulated middle cerebral artery occlusion-induced gut microbiota disorder and the correlated metabolites

**FIGURE 8:**
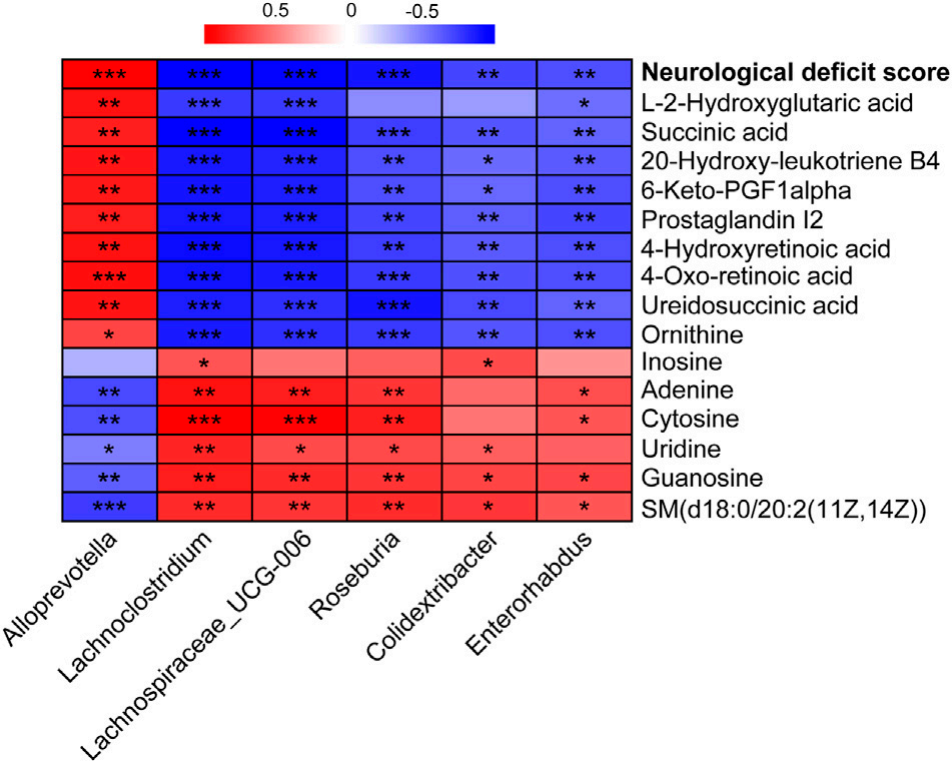
The correlation between the altered genera and metabolites and between the altered genera and neurological deficit score. Correlations were obtained using Spearman test. Red represents positive correlation; blue represents negative correlation. ^*^ *p* < 0.05, ^**^ *p* < 0.01, ^***^ *p* < 0.001.

To reveal the intrinsic relationship between the altered metabolites and gut microbiota, the significantly correlated metabolites and microbes were extracted from the three groups. Spearman’s correlation analysis was applied to represent the covariation between the perturbed metabolites and the gut microbial genera (Figure 8). *Lachnoclostridium*, *Lachnospiraceae_UCG-006*, *Roseburia*, *Colidextribacter*, and *Enterorhabdus* showed similar metabolite relationship patterns, which were positively correlated with guanosine and SM(d18:0/20:2(11Z, 14Z)), but negatively correlated with succinic acid, 20-hydroxy-leukotriene B4, 6-keto-PGF1alpha, prostaglandin I2, 4-hydroxyretinoic acid, 4-oxo-retinoic acid, ureidosuccinic acid, and ornithine. *Alloprevotella* behaved in the opposite manner. Notably, *Lachnoclostridium* and *Colidextribacter* were positively correlated with the inosine levels. *Lachnoclostridium*, *Lachnospiraceae_UCG-006*, *Roseburia*, and *Colidextribacter* were positively correlated with uridine, whereas *Alloprevotella* was negatively correlated. Moreover, *Lachnospiraceae_UCG-006*, *Roseburia*, *Colidextribacter*, and *Enterorhabdus* were positively correlated with guanosine levels. In contrast, *Alloprevotella* was negatively correlated with guanosine levels. It is worth noting that inosine, uridine, and guanosine were significantly restored in the ANP group compared with the model group (Figure 7C). Additionally, a strong correlation was observed between these microbial genera and neurological deficit score. Specifically, *Alloprevotella* was positively correlated with neurological deficit score, while *Lachnoclostridium*, *Lachnospiraceae_UCG-006*, *Roseburia*, *Colidextribacter*, and *Enterorhabdus* were negatively correlated, indicating that the effects of ANP on gut microbiome were associated with attenuated stroke injury.

## 4 Discussion

Accumulating evidence highlights the central role of the microbiota-gut-brain axis in preventing and treating stroke, implying that modulation of the gut microbiota is becoming a new target for anti-stroke therapies. In this study, we validated the therapeutic effects of ANP in a mouse model of acute ischemic stroke using multiple histopathological examinations and a neurological deficit score assessment in mice, and our findings were consistent with clinical data (Guo et al., 2014; Liu et al., 2019). More importantly, we demonstrated that restoring gut microbiota dysbiosis and its metabolite disorder may be one of the main mechanisms of ANP amelioration of ischemic stroke. Particularly, ANP improved the dominant microbial population in MCAO mice, including correction of the increased abundance of the phylum *Bacteroidota*, the family *Prevotellaceae*, and the genus *Alloprevotella*, and restoration of the decreased abundance of the phylum *Firmicutes*, the family *Lachnospiraceae*, and the genera *Lachnoclostridium*, *Enterorhabdus*, *Roseburia*, *Lachnospiraceae_UCG-006*, and *Colidextribacter*. Microbial metabolites related to inflammation and neuroprotection, such as prostaglandin I2 and uridine, were also regulated by ANP treatment.

Overgrowth of *Bacteroidota* (synonym *Bacteroidetes*) is a typical feature of gut microbiota dysbiosis after ischemic stroke, which has been demonstrated in monkeys (Chen et al., 2019c), mice (Singh et al., 2016), and rats (Zhang et al., 2020). A correlation between excessive *Bacteroidota* growth and increased plasma inflammatory cytokine levels was also revealed (Chen et al., 2019c), whereas the systemic inflammatory response that follows a stroke affects infarct volume and neurological function, implying a relationship between *Bacteroidota* and stroke. Although a clinical study reported that *Bacteroidota* levels were decreased in the fecal samples collected from patients with acute ischemic stroke and transient ischemic attack during the first 48 h after admission (Yin et al., 2015), the deregulation of *Bacteroidota* abundance after ischemic stroke has been confirmed in both humans and animals (Singh et al., 2016; Chen et al., 2019c; Xiang et al., 2020; Zhang et al., 2020). In this study, ANP treatment significantly renormalized the disturbance of *Bacteroidota* induced by MCAO modeling, indicating that *Bacteroidota* is a target of ANP intervention. *Firmicutes* are the main bacterial phylum in the mammalian gut. Moreover, the abundance of *Firmicutes* decreased in patients with post-stroke cognitive impairment (Ling et al., 2020) and in MCAO pigs (Jeon et al., 2020), rats (Xian et al., 2022), and mice (Gao et al., 2021). *Firmicutes* showed a negative correlation with lesion volume, midline shift, and hemorrhage volume in ischemic stroke (Jeon et al., 2020). Consistently, this phenomenon was also observed in MCAO mice in this study, while ANP treatment significantly increased *Firmicutes* abundance, suggesting that targeting *Firmicutes* might be one of the mechanisms of ANP against ischemic stroke.

*Prevotellaceae* is a family member of the phylum *Bacteroidota*, and *Alloprevotella* is a genus of bacteria belonging to the family *Prevotellaceae*. Consistent with the alterations in *Bacteroidota*, ANP reversed the increases in *Prevotellaceae* and *Alloprevotella* induced by MCAO modeling, which agrees with the results reported previously in MCAO rats (Li et al., 2020). This demonstrated that the expanded representation of *Prevotellaceae* could be considered a manifestation of gut inflammation (Elinav et al., 2011), indicating that ANP might inhibit the systemic inflammatory response that develops after a stroke. Moreover, *Alloprevotella* was the dominant bacterium in the model group in the LEfSe analysis. Although a previous study found that the combination of Pueraria and Chuanxiong could enrich *Alloprevotella* in the gut of MCAO rats, this bacterium was not identified as a biomarker in the model group (Chen et al., 2019a), the opposite of what we observed in this study. This contradictory result may be due to the effects of different drugs and diets on gut microbiota. Moreover, enrichment of *Alloprevotella* may be associated with an increase in serum trimethylamine N-oxide (Cheng et al., 2017), and the latter is a potential causative factor of first and recurrent stroke (Nam, 2019). Additionally, the abundance of fecal *Alloprevotella* was negatively correlated with serum total antioxidant activity (Wang et al., 2021), and enhancing antioxidative capacity was found to be beneficial for alleviating cerebral ischemia/reperfusion insult (Huang et al., 2022). All these data indicate that ANP administration can protect against stroke by decreasing the abundance of *Prevotellaceae* and *Alloprevotella* in the gut.

*Lachnospiraceae* is a family of bacteria from the phylum *Firmicutes*, and the genera *Lachnoclostridium*, *Lachnospiraceae_UCG-006*, and *Roseburia* belong to the family *Lachnospiraceae*. Deregulation of *Lachnospiraceae* and *Roseburia* abundance after MCAO has been demonstrated in mice (Xia et al., 2019; Gao et al., 2021). Compared with the model group, the abundance of these bacteria was significantly increased after ANP treatment in the present study, consistent with the changes in *Firmicutes*. *Lachnospiraceae* is a butyrate-producing bacterium, and a low abundance of *Lachnospiraceae* is associated with a high risk of stroke (Zeng et al., 2019), implying that ANP treatment may increase butyrate production to protect against stroke. Similarly, *Roseburia* is a well-known butyrate-producing bacterium, and a clinical report showed that enrichment of *Roseburia* may play a protective role in stroke progression and outcome (Gu et al., 2021). As gut microbiota-derived SCFAs such as butyrate may benefit for poststroke recovery, future targeted metabolomic studies that measure SCFAs concentrations are required to confirm this. Additionally, ANP reversed MCAO-induced changes in the abundances of *Lachnoclostridium*, *Enterorhabdus*, *Lachnospiraceae_UCG-006*, and *Colidextribacter.* Although a few studies have reported the functions of *Lachnoclostridium*, *Enterorhabdus*, *Lachnospiraceae_UCG-006*, and *Colidextribacter* in stroke, these four genera, as well as *Roseburia*, showed similar metabolite relationship patterns (Figure 8), indicating that they might play important roles in the prevention of ischemic stroke by ANP.

Bacterial metabolites are the molecular basis for the crosstalk between the host and its microbiota (Benakis et al., 2020). In this study, Spearman analysis revealed a strong association between the gut microbiota and their metabolites in response to ANP treatment. Generally, the gut microbiota affects metabolites, including eicosanoids (prostaglandin I2, 20-hydroxy-leukotriene B4, and 6-keto-PGF1alpha) and nucleosides (uridine, guanosine, and inosine). Moreover, *Lachnoclostridium*, *Lachnospiraceae_UCG-006*, *Roseburia*, *Colidextribacter*, and *Enterorhabdus* showed similar metabolite relationship patterns, whereas *Alloprevotella* behaved in the opposite manner. These metabolites are affected by several metabolic pathways, including those of pyrimidine, arachidonic acid, and purine metabolism. In the pyrimidine metabolism pathway, ANP increased the levels of uridine and cytosine and decreased the abundance of ureidosuccinic acid. Uridine can be used by the brain and may be the major precursor for salvage pathways of pyrimidine nucleotides (Cansev, 2006). It has been shown that uridine can decrease the edema in the hippocampus and striatum caused by traumatic brain injury (Kabadi and Maher, 2010) and increase the formation of brain synapses (Wurtman et al., 2010).

Notably, guanosine and inosine, which are involved in purine metabolism, are reportedly neuroprotective. Guanosine exhibits anti-oxidative and anti-inflammatory properties in cerebral ischemic injury (Dal-Cim et al., 2011; Dal-Cim et al., 2013), while inosine can improve behavioral outcomes after stroke (Chen et al., 2002; Heuss, 2002; Smith et al., 2007). Therefore, our findings suggest that uridine, guanosine, and inosine may be potential neuromodulators produced by certain genera that were increased in the ANP group. Uridine may be produced by *Lachnoclostridium*, *Lachnospiraceae_UCG-006*, *Roseburia*, and *Colidextribacter*. Guanosine may be produced by *Lachnoclostridium*, *Lachnospiraceae_UCG-006*, *Roseburia*, *Colidextribacter*, and *Enterorhabdus*, while inosine may be produced by *Lachnoclostridium* and *Colidextribacter*.

Moreover, ANP also decreased the levels of prostaglandin I2, 20-hydroxy-leukotriene B4, and 6-keto-PGF1alpha. These metabolites are involved in arachidonic acid metabolism, an essential pathway involved in inflammatory reactions. Prostaglandins and leukotrienes are downstream products of arachidonic acid. Cyclooxygenase catalyzes the formation of prostaglandins from arachidonic acid, and lipoxygenase catalyzes the formation of leukotrienes from arachidonic acid (Shoeb et al., 2011). ANP reduced the levels of prostaglandin I2, 20-hydroxy-leukotriene B4, and 6-keto-PGF1alpha, indicating anti-inflammatory activity. Prostaglandin I2, 20-hydroxy-leukotriene B4, and 6-keto-PGF1alpha showed similar trends against gut microbiota patterns, being positively correlated with *Alloprevotella*, but negatively correlated with *Lachnoclostridium*, *Lachnospiraceae_UCG-006*, *Roseburia*, *Colidextribacter*, and *Enterorhabdus*. Notably, succinic acid was affected in several metabolic pathways, including the cAMP signaling pathway, alanine, aspartate, and glutamate metabolism, lysine degradation, and GABAergic synapses. The accumulation of succinic acid is a universal metabolic signature of ischemic tissues, and the inhibition of ischemic succinic acid accumulation is a potential therapeutic target (Chouchani et al., 2014), which is consistent with ANP treatment (Supplementary Figure S5). These phenomena indicate crosstalk between the metabolites in the brain and those produced by the gut microbiota.

Notably, traditional Chinese medicine (TCM) has multiple and complex components, and “multiple components against multiple targets” is constantly being proposed as the therapeutic principle of TCM (Xu et al., 2017). ANP is one of the most famous traditional Chinese patent medicines, comprising multiple Chinese medicinal materials, and is arguably beneficial because it produces a synergistic effect from its multiple components for the treatment and prevention of stroke. According to previous reports, the neuroprotective effect of ANP might be attributed to reduction of the Bax/Bcl-2 ratio and the caspase-3 level (Wang et al., 2014), inhibition of matrix metalloproteinase and protection of tight junction proteins (Tsoi et al., 2019), or activation of the glycogen synthase kinase 3-β/heme oxygenase-1 pathway in brain tissue (Zhang et al., 2021). The modulation of gut microbiota we focus on here might be another important factor in the neuroprotective effect of ANP. We believe that it is through the synergistic effect of multiple targets that ANP can exert a beneficial action on the ischemic brain.

## 5 Conclusion

The results of our study not only demonstrate that ANP significantly attenuates cerebral ischemia/reperfusion injury in mice but also provide mechanistic insights into the protective actions of ANP against acute ischemic stroke, with a specific emphasis on the potential roles of several gut bacteria such as the phyla *Bacteroidetes* and *Firmicutes*, the families *Lachnospiraceae* and *Prevotellaceae*, and the genera *Alloprevotella* and *Roseburia*. Moreover, a close link between metabolites related to inflammation (prostaglandin I2, 20-hydroxy-leukotriene B4, and 6-keto-PGF1alpha) and neuroprotection (uridine, guanosine, and inosine) and certain genera, including *Alloprevotella*, *Lachnoclostridium*, *Lachnospiraceae_UCG-006*, *Roseburia*, *Colidextribacter*, and *Enterorhabdus*, was also identified in ischemic stroke. Future studies should employ fecal transplantation to validate the beneficial effect of ANP on gut microbiota modulation in stroke. Clinical studies are warranted to assess the therapeutic potential of these important bacteria better, as revealed by ANP.

## Data Availability

The datasets presented in this study can be found in online repositories. The names of the repository/repositories and accession number(s) can be found below: https://www.ncbi.nlm.nih.gov/, PRJNA855110.
