# Supplementary material for: Angong Niuhuang Pill ameliorates cerebral ischemia/reperfusion injury in mice partly by restoring gut microbiota dysbiosis
Source: Front Pharmacol. 2022 Sep 15;13:1001422. doi: 10.3389/fphar.2022.1001422 (PMC9520595; doi:10.3389/fphar.2022.1001422)
Supplement: Supplementary file 3 [file DataSheet1.docx]

Supplementary Materials

# Supplementary Methods

## HPLC analysis of ANP

ANP (3.0 g) was minced with scissors and ultrasonically extracted with 30 mL ethyl alcohol and ultrapure water (7/3, v/v) for 2 h. After centrifugation (4200 rpm, 10 min), the supernatant was collected and made up to a final volume of 30 mL with ethyl alcohol and ultrapure water (7/3, v/v). Then, a volume of 100 μL supernatant was added to 1900 μL methanol, and the mixture was used for HPLC analysis after vortex and centrifugation (12000 rpm, 10 min).

HPLC analysis was executed on a Zorbax SB-C18 column (250 × 4.6 mm, 5 μm) (Agilent, Santa Clara, CA, United States) using an ACQUITY ARC HPLC system (Waters Corporation, Milford, MA, United States) combined with a 2998 photo diode array (PDA) system (Waters Corporation). The HPLC conditions were set as follows: detection wavelength, 254 nm; column temperature, 30 °C; flow rate, 0.8 mL/min; injection volume, 10 μL; mobile phases A, ultrapure water containing 0.1% formic acid; mobile phases B, acetonitrile. The gradients were as follows: 15%-22%B, 0-10 min; 22%-22%B. 10-20 min; 22%-30%B, 20-30 min; 30%-60% B, 30-50 min. The HPLC chromatograms of ANP was recorded and the main peaks were identified by comparing with the reference standards.

## The detailed parameters for LC-MS analysis

The liquid chromatography conditions were set as follows: column temperature, 45 °C; flow rate, 0.35 mL/min; injection volume, 2 μL; mobile phases A, water containing 0.1% formic acid; mobile phases B, acetonitrile containing 0.1% formic acid. The gradients were as follows: 5%B, 0-2 min; 5%-30%B, 2-4 min; 30%-50%B, 4-8 min; 50%-80%B, 8-10 min; 80%-100%B, 10-14 min; 100%B, 14-15 min; 100%-5%B, 15-15.1 min; 5%B, 15.1-16 min.

Positive and negative ion mass spectra were acquired, and the mass spectrometry conditions were as follows: mass range, *m/z* 100-1200; capillary temperature, 320 ℃; aux gas heater temperature, 350 ℃; spray voltage, 3800 V (positive) / -3000 V (negative); sheath gas flow rate, 40 Arb (positive) / 35 Arb (negative); aux gas flow rate, 10 Arb (positive) / 8 Arb (negative).

## The detailed parameters for GC-MS analysis

The gas chromatography conditions were set as follows: carrier gas, helium; flow rate, 1 mL/min; injection volume, 1 μL. The gradients were as follows. The initial oven temperature was 60 ℃ (held for 0.5 min), ramped to 210 ℃ at the rate of 8 ℃/min, to 270 ℃ at the rate of 15 ℃/min, to 305 ℃ at the rate of 20 ℃/min, and finally oven temperature was 305 ℃ (held for 5 min).

The mass spectrometry conditions were put as follows: ion source temperature, 230 ℃; quadrapole temperature, 150 °C; collision energy, 70eV; mass range, *m/z* 50-500.


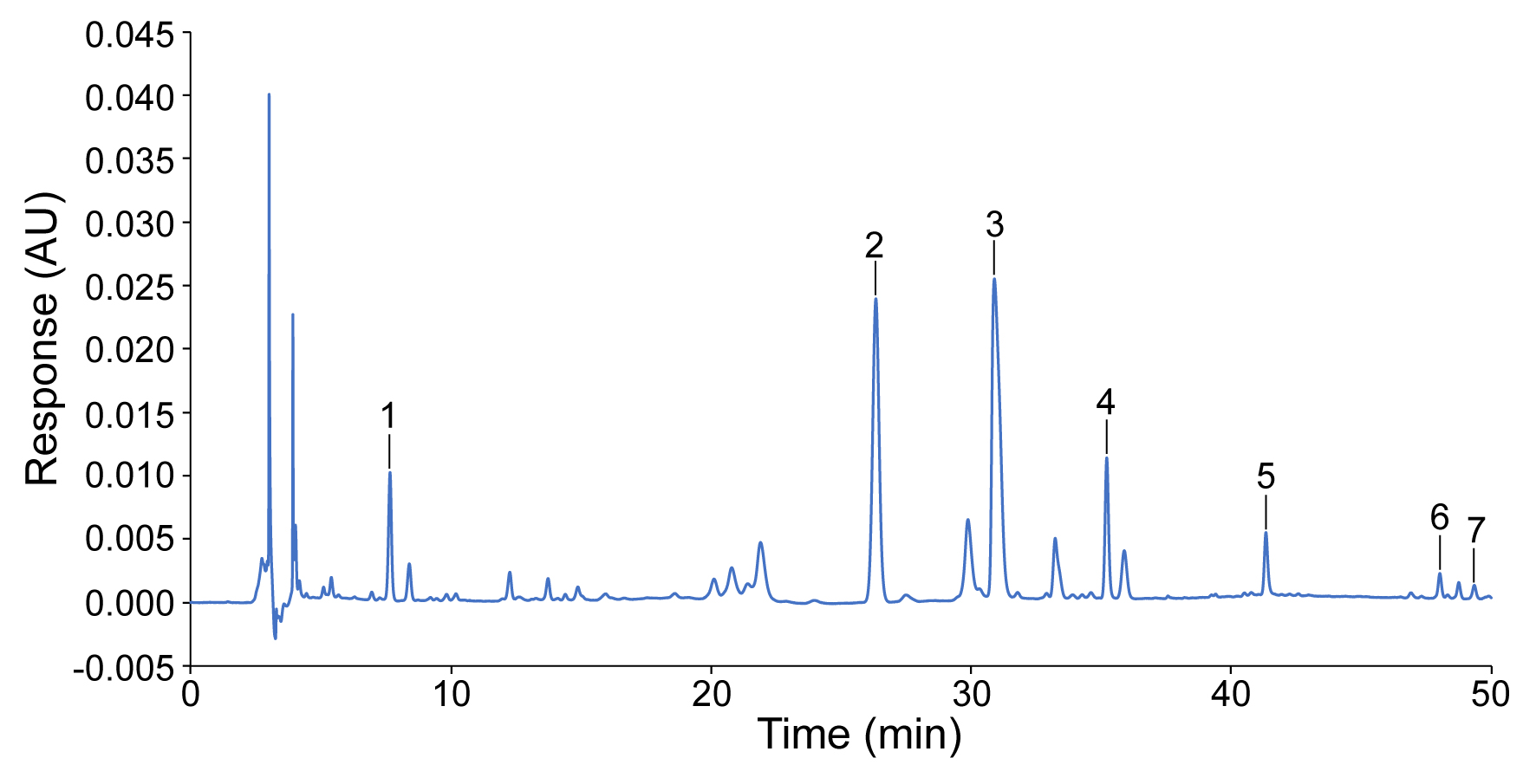


Supplementary Fig. 1 The HPLC profile of ANP. Seven chromatographic peaks were identified: (1) geniposide, (2) baicalin, (3) berberine, (4) wogonoside, (5) baicalein, (6) wogonin, (7) oroxylin A.


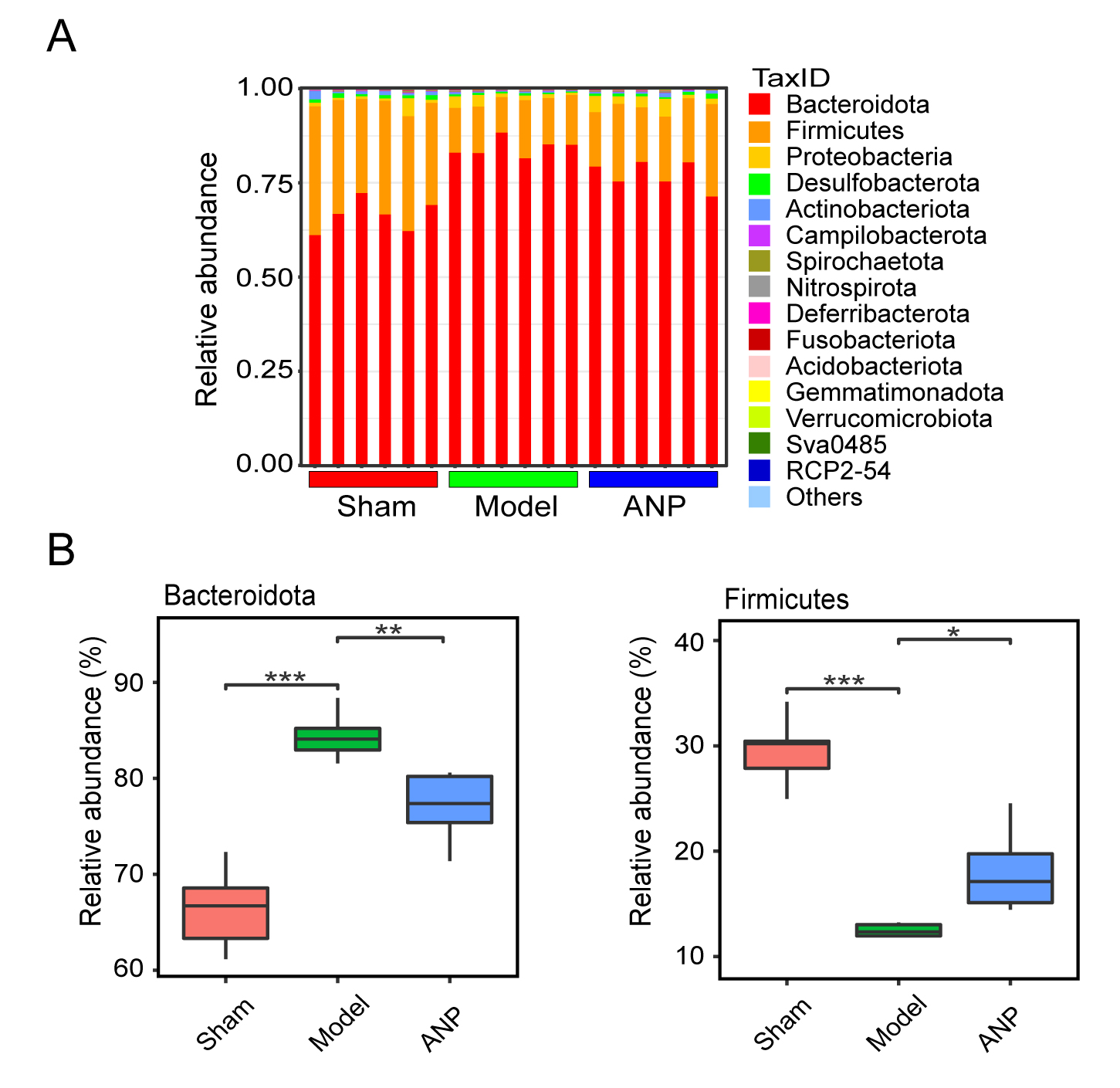


Supplementary Fig. S2 ANP regulated the gut microbiota at the phylum level.

(A) The gut microbiota composition at the phylum level. (B) The statistical analysis at the phylum level. ^*^*P* < 0.05, ^**^*P* < 0.01, ^***^*P* < 0.001.


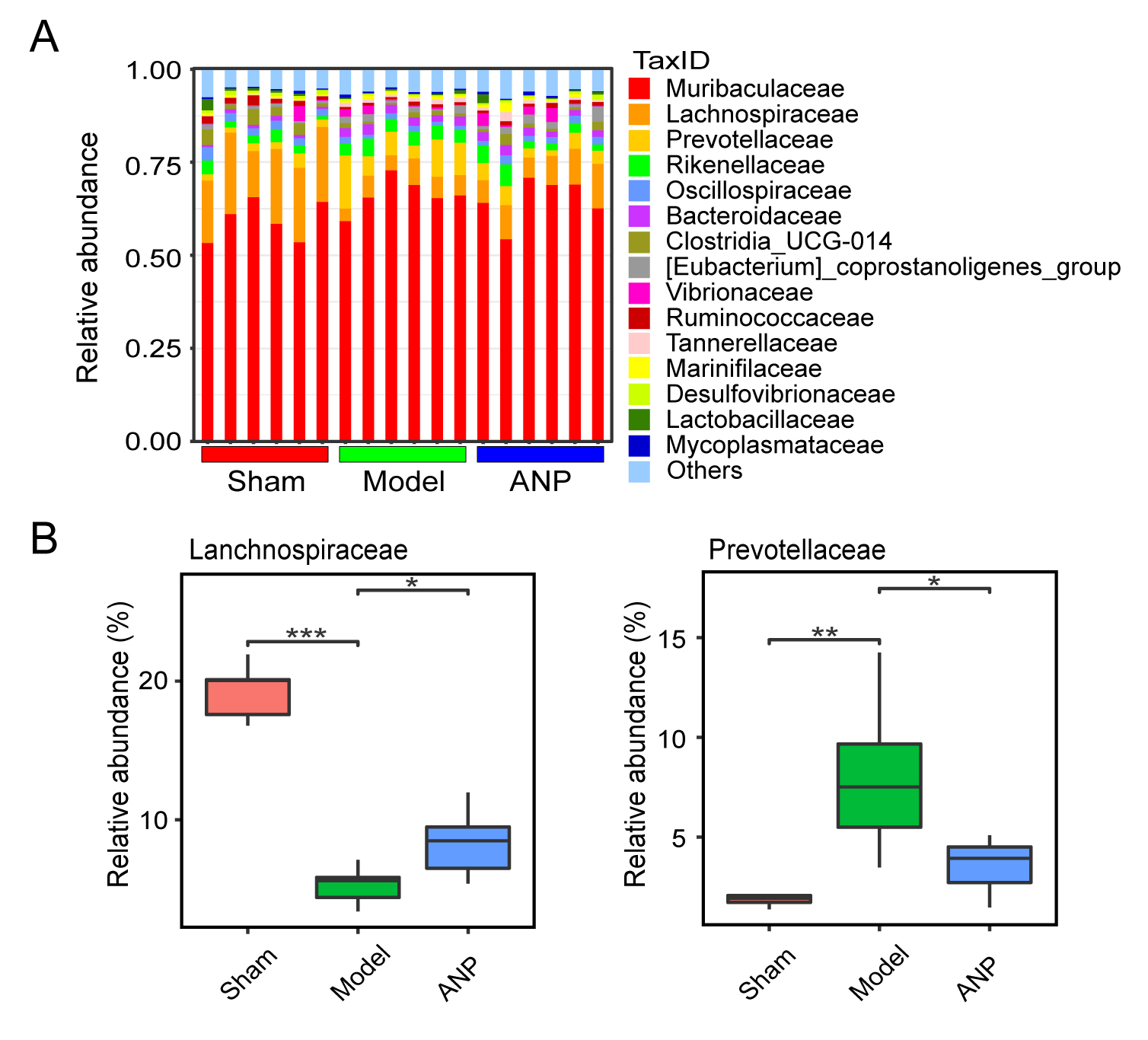


Supplementary Fig. S3 ANP regulated the gut microbiota at the family level.

(A) The gut microbiota composition at the family level. (B) The statistical analysis at the family level. ^*^*P* < 0.05, ^**^*P* < 0.01, ^***^*P* < 0.001.


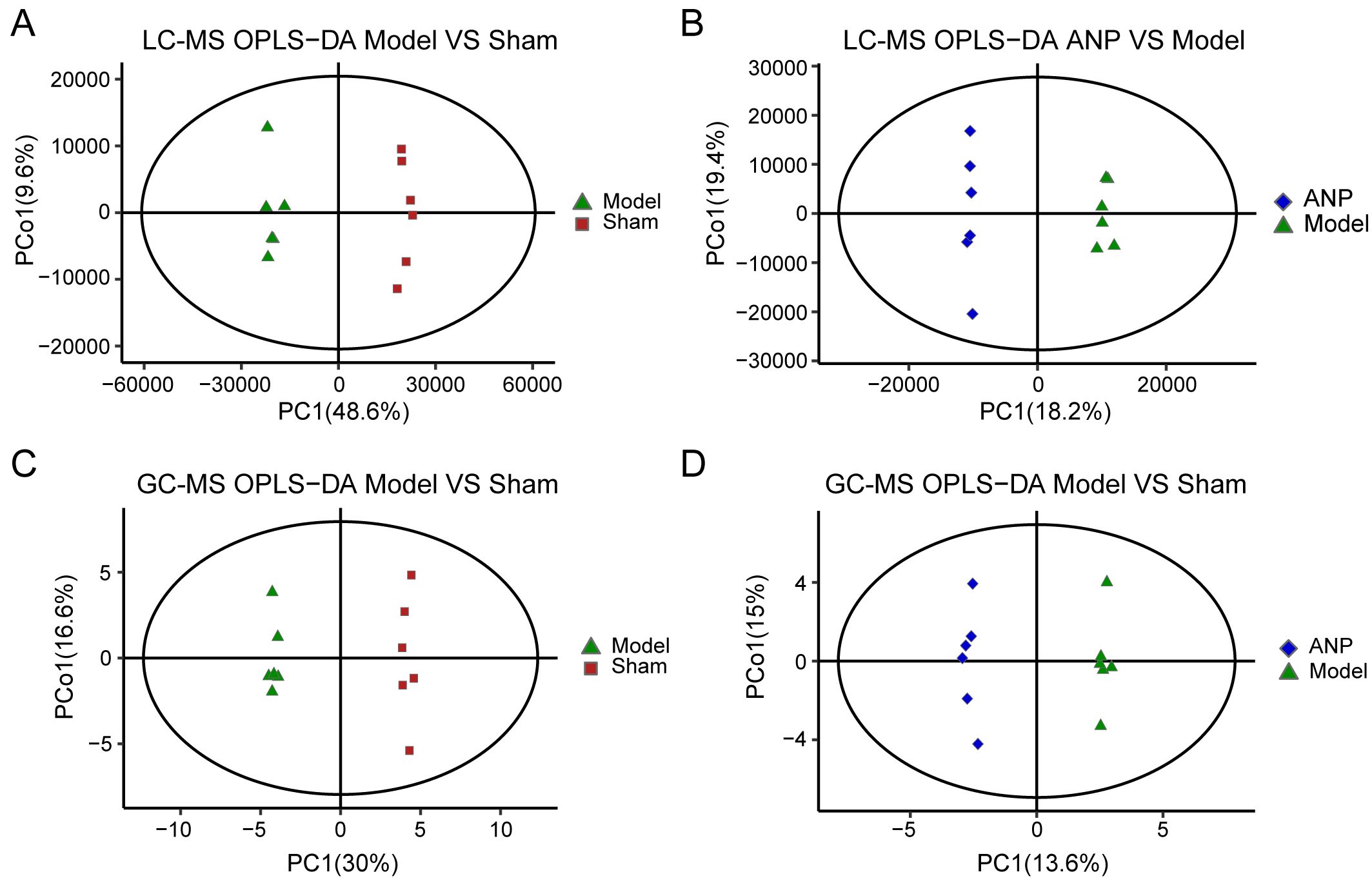


Supplementary Fig. S4 The OPLS-DA score plots.

(A) The OPLS-DA score plot between the model group and the sham group based on the metabolomic data from LC-MS analysis. (B) The OPLS-DA score plot between the ANP group and the model group based on the metabolomic data from LC-MS analysis. (C) The OPLS-DA score plot between the model group and the sham group based on the metabolomic data from GC-MS analysis. (D) The OPLS-DA score plot between the ANP group and the model group based on the metabolomic data from GC-MS analysis.


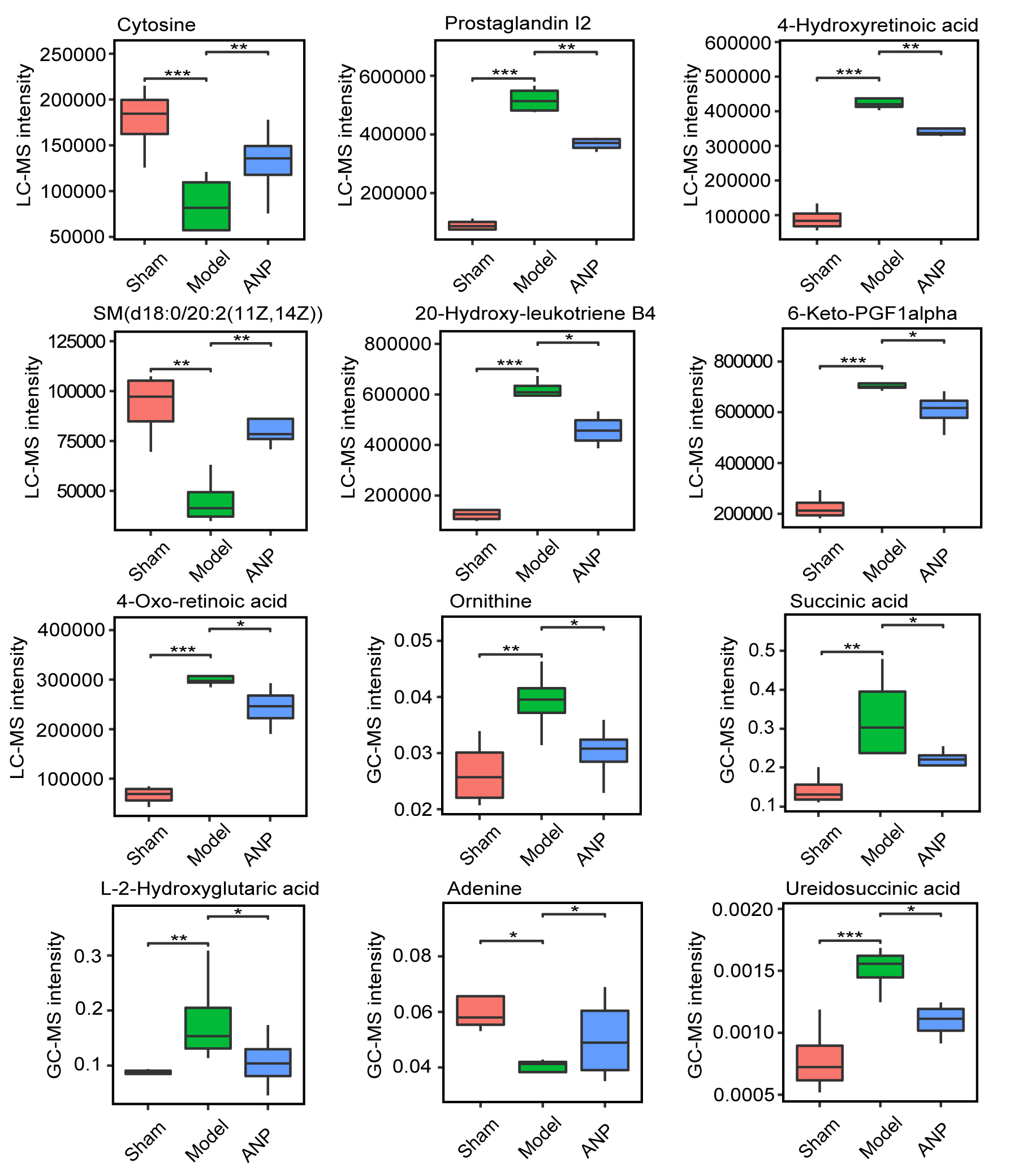


Supplementary Fig. S5 The statistical analysis of the key differentially expressed metabolites. ^*^*P* < 0.05, ^**^*P* < 0.01, ^***^*P* < 0.001.
